# Sustainable Encapsulation of Biocontrol Agents: Cross-Linker Influence on Carboxymethylcellulose-Based Microbeads

**DOI:** 10.1021/acsomega.5c06970

**Published:** 2026-03-13

**Authors:** Mayté P. Zaldivar, Jean Carlos F. Machado, Lívia C. Massimino, Marcel S. Marques, José Eduardo M. de Almeida, Ana Paula S. Bartels, Ricardo Bortoletto-Santos, Hernane da S. Barud

**Affiliations:** † Biopolymers and Biomaterials Laboratory (BioPolMat), 74374 University of Araraquara (UNIARA), 1217 Carlos Gomes Street, Downtown, Araraquara, São Paulo 14801-340, Brazil; ‡ Postgraduate Program in Environmental Technology, 42496 University of Ribeirão Preto (UNAERP), Avenida Costábile Romano, 2201—Nova Ribeirânia, Ribeirão Preto, São Paulo 14096-900, Brazil; § APTA/SAA-SP, Biological Institute, Alameda dos Vidoeiros, 1097—Sítios de Recreio Gramado, Campinas, São Paulo 13101-680, Brazil

## Abstract

The use of biopolymers for encapsulating active ingredients is a well-established approach, with ionotropic gelation representing a viable technique. This method allows the use of various cross-linking agents, though the physicochemical properties of the resulting materials can vary depending on the cross-linker selected. This study aimed to evaluate calcium (Ca^2+^) and aluminum (Al^3+^) ions as cross-linkers for the formation of carboxymethylcellulose (CMC) microbeads that can carry biological agents. Following comparative analyses, the most effective cross-linker was used for encapsulating the entomopathogenic fungus *Beauveria bassiana* strain IBCB66. Encapsulation of *B. bassiana* within a biopolymer bead matrix was found to be a promising strategy to preserve its biological control properties. Beads cross-linked with Al^3+^ (CMC_Al^3+^) demonstrated superior thermal stability (*T*_max of 165.76 and 386.71 °C) and swelling capacity (≈800%) compared to those cross-linked with Ca^2+^ (*T*_max of 211.78, 223.22, 309.29, and 368.95 °C, and swelling capacity of ≈200%). CMC_Al^3+^ beads also exhibited a uniform average size (1.92 ± 0.11 mm), in contrast to the heterogeneous conglomerates observed in CMC_Ca^2+^ beads. Blastospores of *B. bassiana* were efficiently encapsulated in CMC_Al^3+^ beads via a simple and rapid method, with 85% germination observed on the bead surface after five months of storage at −18 °C. These findings indicate that aluminum is a promising cross-linking agent for CMC-based encapsulation matrices in biological control applications.

## Graphical abstract

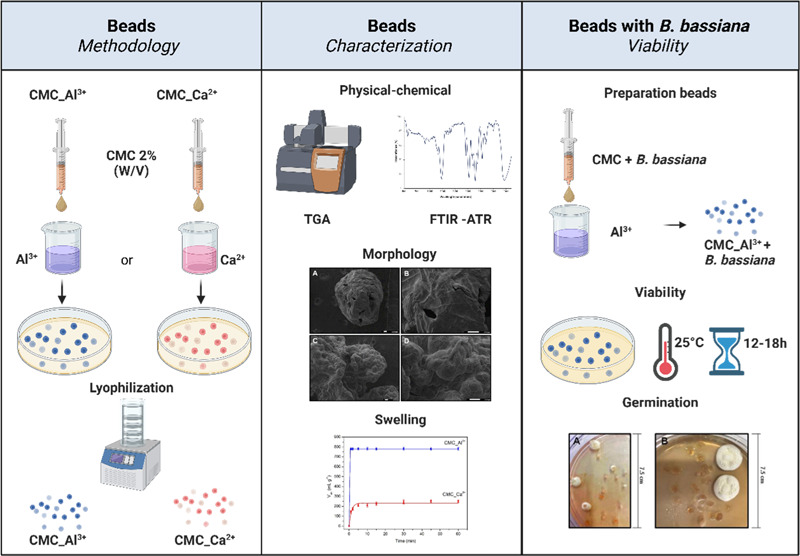

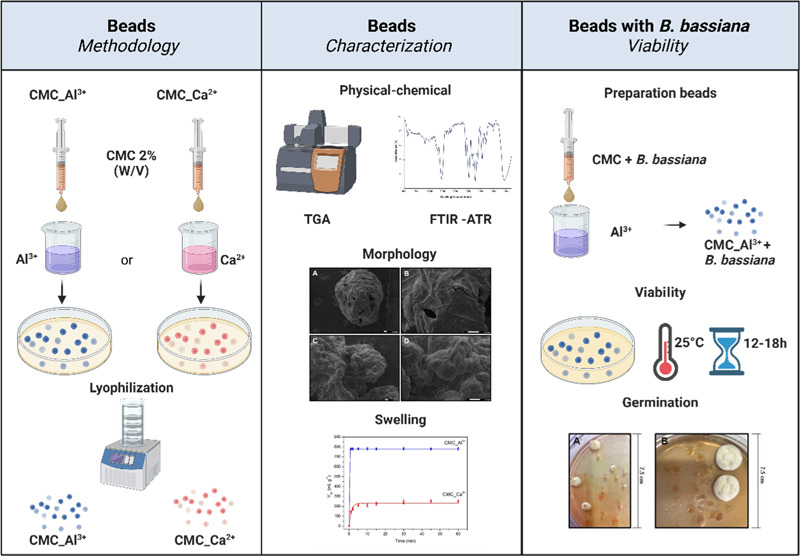

## 1 Introduction

Biopolymers are derived from natural sources, either directly from living organisms or via chemical synthesis from biological materials.^1^ These materials can be structured at macro-, micro-, and nanoscales and are widely used across various industries, including food, pharmaceuticals, cosmetics, personal care, and agriculture.^2,3^ Due to their biodegradability and biocompatibility, they have garnered increasing attention as sustainable alternatives to synthetic polymers.

Cellulose is the most abundant natural polysaccharide, and it has limited direct applications due to its water insolubility.^4^ However, chemical modifications yield cellulose derivatives with enhanced solubility in aqueous and organic solvents. Carboxymethylcellulose (CMC) is one of the most commonly used water-soluble derivatives. CMC is a biodegradable, biocompatible, nontoxic, and low-cost anionic polymer with broad industrial applications in food, medicine, agriculture, and personal care products.^5,6^ Moreover, CMC can form hydrogel particles through chemical or physical cross-linking.^7^

Hydrogels are three-dimensional polymeric networks with a strong affinity for water, capable of retaining large volumes without losing structural integrity.^7,8^ Physical cross-linking of CMC is often achieved using multivalent metal ions through ionotropic gelation, a process that relies on electrostatic interactions between the metal ions and functional groups (−COO^–^ and −OH) on the CMC backbone.^4,9,10^ This method is simple, rapid, cost-effective, energy-efficient, and environmentally friendly, making it suitable for large-scale applications.^4,10^ However, it may present limitations, such as uneven ion distribution and uncontrollable coordination rates.^10^ The hydrogel-forming ability of CMC makes it suitable for use in encapsulation technologies, particularly for delivering and protecting bioactive compounds.^6,11^ Encapsulation involves enclosing active materials, such as liquids or solids, within polymeric matrices that can release their contents in a controlled manner.^12^ This technique is widely applied for incorporating enzymes, cells, or microorganisms, including entomopathogenic fungi.^12^

*Beauveria bassiana* is one of the most explored entomopathogenic fungi in biological control programs for insect pests of different crops worldwide.^13^ It acts as a parasite causing the white muscardine disease in various arthropod species like *Diptera*,^14,15^ *Coleoptera*,^16^ *Hemiptera*,^17^ *Lepidoptera*,^18^ *Isoptera*,^19^ *Orthoptera*,^20^ *Thysanoptera*,^21^ and *Homoptera*.^22^ This fungus does not need to be consumed by insects because it can infect them by contact. It exhibits high virulence and pathogenicity variation without compromising human health.^23^ One of the main challenges limiting the broader application of entomopathogenic fungi in biological control is maintaining their viability during long-term storage.

To address this, the development of effective formulations is crucial for extending shelf life and ensuring field efficacy. Encapsulation has emerged as a promising strategy to protect fungal propagules from environmental stressors, such as temperature fluctuations, photodegradation, and oxidation, while also enabling controlled release.^24^ Furthermore, encapsulation facilitates the handling and application of fungal agents in the form of dry biopolymeric beads or granules, enhancing their distribution in soil.^24–27^ Although alginate has been widely used as the primary matrix for fungal encapsulation,^24–27^ studies involving carboxymethylcellulose (CMC) are limited,^28,29^ with few reports exploring its use as the main encapsulating agent for entomopathogenic fungi. Interestingly, *B. bassiana* has been shown to degrade CMC via cellulase production and can utilize it as a carbon source, suggesting its potential compatibility with CMC-based encapsulation systems. On the other hand, ionotropic gelation of carboxymethylcellulose (CMC) with multivalent cations is well established and has been revisited in recent years for beads and hydrogels produced with Ca^2+^ or Al^3+^. Broad reviews on CMC gels describe metal-ion cross-linking routes and how counterion valence governs network formation and properties (i.e., swelling, mechanics, and thermal behavior).^30^ In addition, recent studies report CMC hydrogel/bead systems formed in AlCl_3_ solutions, often achieving high water uptake and robust networks, while Ca^2+^ continues to be widely used to obtain physically cross-linked CMC hydrogels and Ca-exchanged CMC powders that self-gel in water.^11,31^ Consistent with the polyanion gelation literature, trivalent ions, such as Al^3+^, typically yield denser, less swellable, yet mechanically stronger ionic networks compared to divalent Ca^2+^, due to their higher charge density and multidentate coordination.^32^ Thus, applications targeting the encapsulation of entomopathogenic fungi remain scarce relative to alginate-based matrices, which dominate the field.^2^

In this context, this study successfully synthesized and characterized carboxymethylcellulose (CMC) beads via ionotropic gelation using Ca^2+^ and Al^3+^ ions, identified the most effective cross-linking agent, and demonstrated the potential application of these beads for encapsulating the entomopathogenic fungus *B. bassiana*.

## 2 Results and Discussion

### 2.1 Morphological Characterization

All CMC beads (CMC_Al^3+^ and CMC_Ca^2+^) were prepared using the ionotropic gelation method. The beads formed instantly when the CMC solution dripped into the reticulating agents.^6^ Figure 1 illustrates the preparation of CMC beads and digital photographs of the spheres after the dripping and drying.

**Figure 1:**
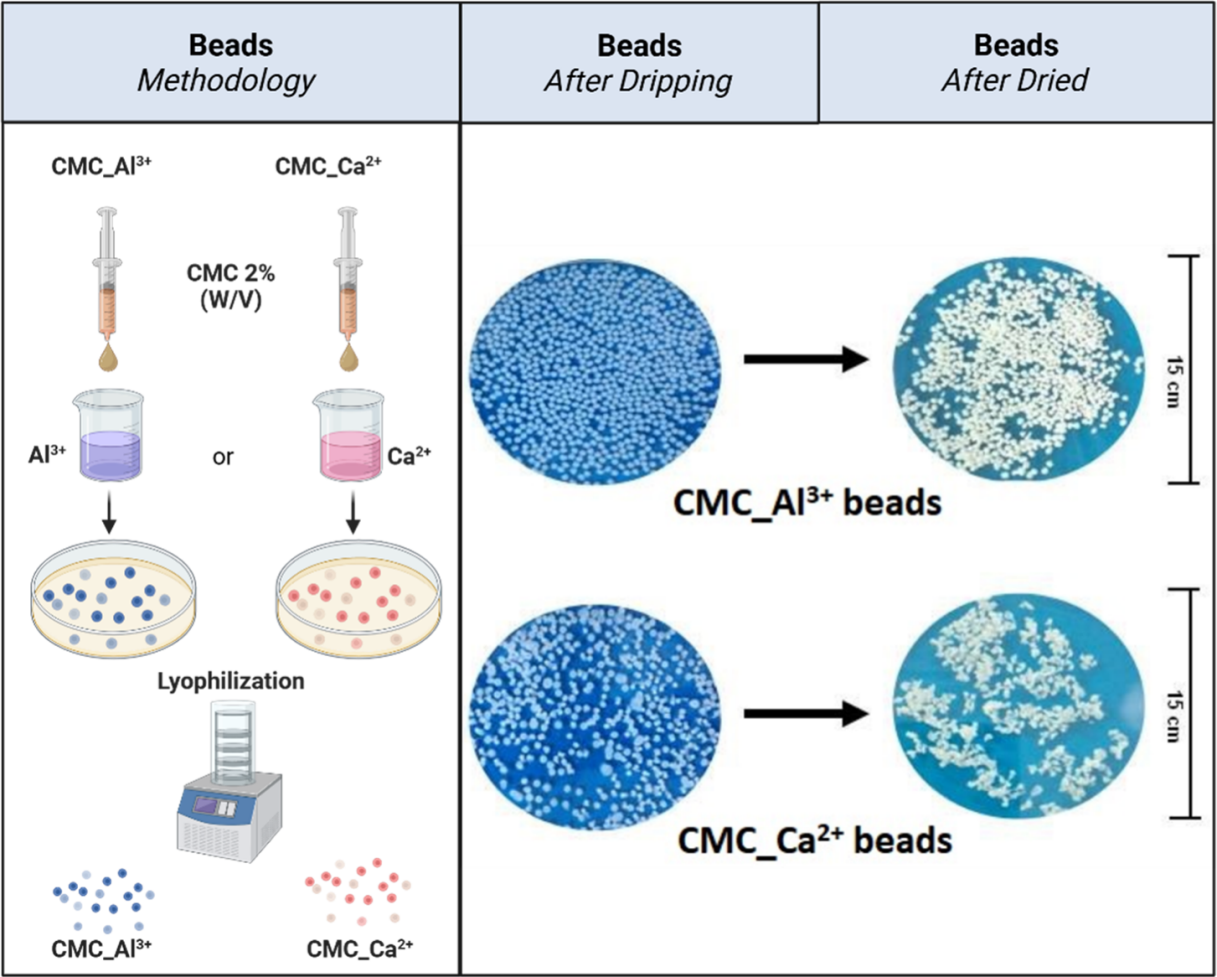
Schematic representation of the CMC beads in the obtention process. Features of CMC beads after the dripping and drying processes. The photographs were taken by the authors. Graphic images were created using BioRender.

The photographs revealed that both AlCl_3_ and CaCl_2_ allowed the initial formation of spherical structures. However, after the drying process, striking differences were observed between the systems. Spheres cross-linked with Ca^2+^ ions presented structural collapse and coalescence effect, resulting in irregular conglomerates of various sizes. In contrast, the spheres formed with Al^3+^ maintained their spherical shape and presented greater morphological uniformity.

This behavior can be attributed to the differences in valence and charge density between the cations. The Al^3+^ ion, with a greater positive charge, forms stronger and denser ionic bridges with the carboxylate groups (−COO^–^) of CMC, favoring the formation of a more compact and deformation-resistant three-dimensional network.^33^ On the other hand, Ca^2+^, due to its lower charge, forms less intense ionic bonds, resulting in a less cross-linked and structurally unstable polymeric matrix against contraction during drying. Furthermore, the rapid diffusion of Ca^2+^ can induce surface gelation, resulting in a rigid outer shell over a still fluid interior, which aggravates the collapse of the structure due to water loss. In this context, the multidentate complexes formed with Al^3+^ provide greater mechanical resistance, preventing deformation and grouping of the spheres. Thus, the results indicate that the use of AlCl_3_ as a cross-linker is more efficient in producing CMC beads with good dimensional stability.

In addition, scanning electron microscopy (SEM) images of CMC beads reticulated with Al^3+^ and Ca^2+^ ions after the drying process are shown in Figure 2. The SEM images revealed a single CMC spherical-like bead obtained from the Al^3+^ reticulation (A), while conglomerates of CMC were obtained with the Ca^2+^ reticulation (B). It was found that the surface of CMC_Al^3+^ beads was slightly rough with some cracks, whereas the CMC_Ca^2+^ conglomerates exhibited a rough and irregular morphology surface.^34^

**Figure 2:**
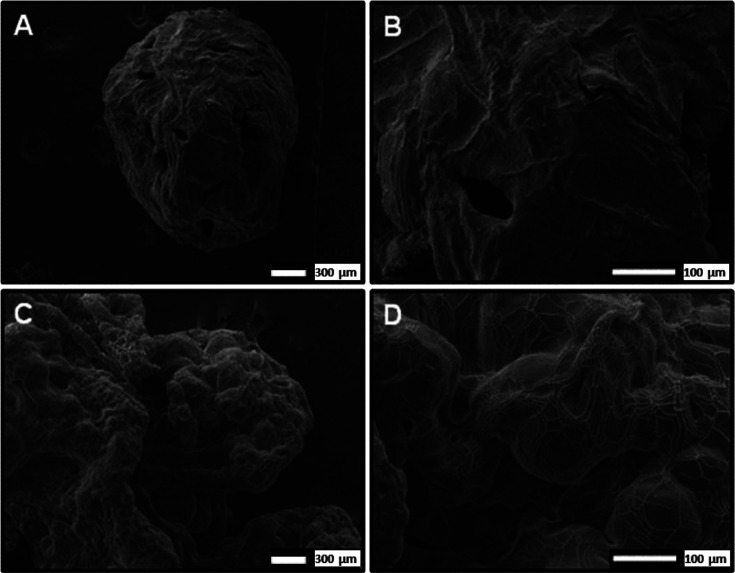
SEM images of CMC beads reticulated with Al^3+^ (A,B) and Ca^2+^ (C,D) ions with different magnifications (40× and 150×, respectively).

The FTIR spectra of not reticulated CMC lyophilized gel (CMC_NR) and the reticulated beads with Al^3+^ (CMC_Al^3+^) and Ca^2+^ (CMC_Ca^2+^) are shown in Figure 3. All spectra displayed the characteristic absorption bands of CMC: a broad band at 3700–3000 cm^–1^ assigned to O–H stretching, asymmetric and symmetric stretching of CH_2_ groups at 2920 and 2870 cm^–1^, the asymmetric and symmetric stretching of the carboxylate anion (COO^–^) at 1583 and 1414 cm^–1^, an in-plane OH bending vibration at 1321 cm^–1^, and C–O/C–O–C stretching at 1105, 1052, and 1020 cm^–1^.^4–35^ Upon cross-linking, notable changes were observed: both CMC_Al^3+^ and CMC_Ca^2+^ spectra showed new bands at 1486 and 1458 cm^–1^, respectively, which are attributed to the coordination of carboxylate groups with Al^3+^ and Ca^2+^ ions. These additional bands indicate the formation of ionic bridges between the metal ions and the carboxylate groups of CMC. Moreover, slight shifts and intensity changes were observed in the COO^–^ stretching region (1583 and 1414 cm^–1^), further confirming the involvement of carboxylate groups in the reticulation process. These spectral changes reflect modifications in the electronic environment and charge distribution of the carboxylate groups upon coordination with the cations, confirming that ionic cross-linking has occurred. In particular, the higher charge density of Al^3+^ promotes stronger electrostatic interactions and a more stable cross-linked network compared with Ca^2+^, which is consistent with the differences observed in bead morphology and swelling behavior. The direct labeling of these bands in Figure 3 highlights the spectral differences and provides clearer evidence of successful cross-linking.^4^

**Figure 3:**
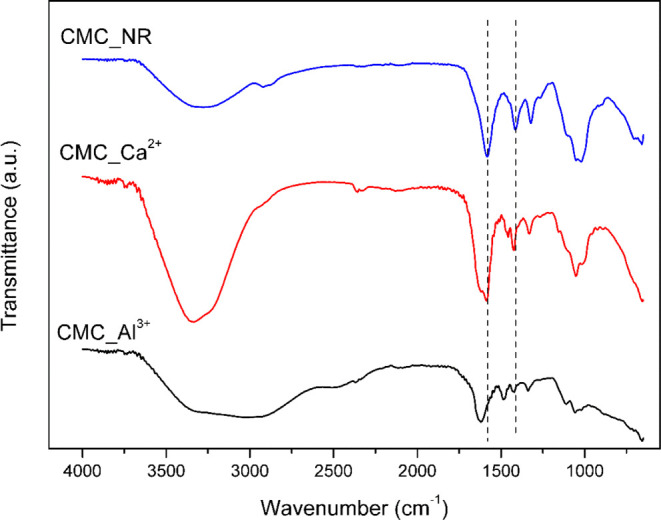
FTIR spectra of nonreticulated CMC gel (CMC_NR) and reticulated beads with Al^3+^ (CMC_Al^3+^) and Ca^2+^ (CMC_Ca^2+^). Newly appearing bands (1486 and 1458 cm^–1^) are labeled and correspond to carboxylate coordination with Al^3+^ and Ca^2+^, respectively.

Thermal properties were studied using simultaneous TGA/DTG/DSC analysis (Figure 4). All curves show multiple degradation stages, and the temperatures of the maximum decomposition rate (*T*_max_) for each stage, as determined by DTG, are shown in Table 1.

**Figure 4:**
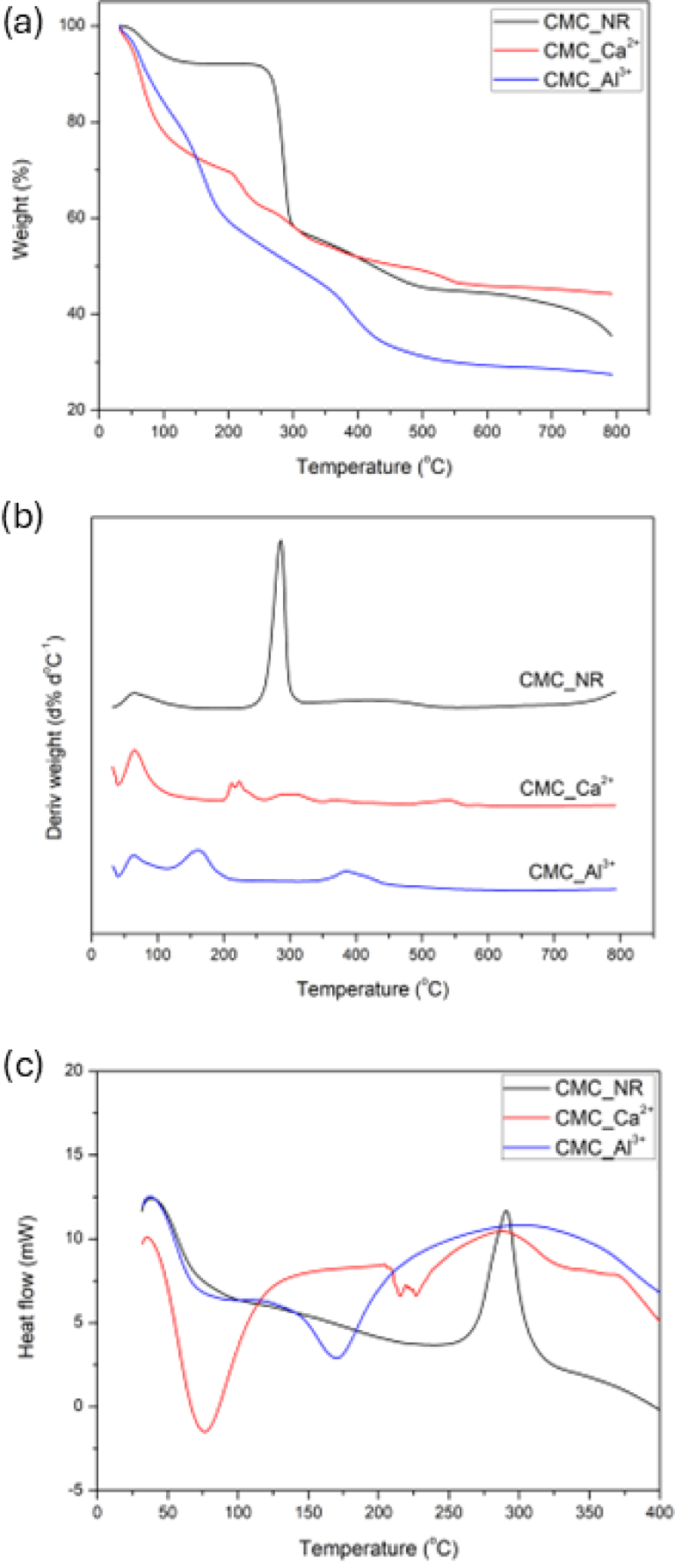
TG (a), DTG (b), and DSC (c) curves of the not-reticulated CMC (CMC_NR) and the reticulated CMC beads with Al^3+^ (CMC_Al^3+^) and Ca^2+^ (CMC_Ca^2+^).

**Table 1:** Decomposition Temperatures of Reticulated (CMC_Al^3+^ and CMC_Ca^2+^) and Not Reticulated (CMC_NR) CMC Materials Obtained by TG/DTG

*T* (°C)	CMC_NR	CMC_Al^3+^	CMC_Ca^2+^
*T*_max_ (1)	64.56	62.54	66.64
*T*_max_ (2)	286.21	165.76	211.78 and 223.22
*T*_max_ (3)	461.80	386.71	309.29
*T*_max_ (4)			368.95
*T*_max_ (5)			539.72

The sample CMC_NR gel shows three degradation stages. The first can be attributed to the release of adsorbed water molecules, and the last one is the decomposition of this polymer, generating a carbonaceous material. The *T*_max_ of 286.21 °C corresponds to the reported values for this polymer. The *T*_max_ value of 461.80 °C could be attributed to a more stable hydrated complex of CMC or the pyrolysis of carboxyl groups. Liu et al. (2015)^33^ reported the pyrolysis of carboxyl groups (at about 700 °C) as another decomposition form for raw CMC.^4^ This process could be affected by the CMC dissolution in water, resulting in a decrease in its *T*_max_ value.

The reticulation process significantly affects the thermal stability of CMC gels when metal ions are used to form cross-linked beads. It was observed that the reticulated beads exhibited lower thermal stability compared to nonreticulated CMC (CMC_NR), which contrasts with the general expectation that cross-linking enhances the thermal resistance of polymeric materials. This reduced stability can be attributed to the replacement of Na^+^ counterions by multivalent cations (Ca^2+^ or Al^3+^), which alters the coordination environment and modifies the degradation pathway,^4^ promoting earlier decomposition stages as reported for other polysaccharide systems.^35^ Moreover, the thermal decomposition of the raw cross-linking agents occurs at relatively low temperatures (*T*_max_ of 178.33 °C for AlCl_3_ and 140.56 °C for CaCl_2_), which may also contribute to the earlier onset of degradation in the reticulated systems. Within the cross-linked samples, CMC_Al^3+^ beads exhibited two main decomposition stages with *T*_max_ at 165.76 and 386.71 °C, while CMC_Ca^2+^ beads showed multiple events at 211.78 °C, 223.22 °C, 309.29 °C, and 368.95 °C. In addition, CMC_Al^3+^ displayed a lower residual mass compared with CMC_Ca^2+^ across almost the entire temperature range, which is likely related to the higher charge density of Al^3+^ leading to more efficient coordination and enhanced depolymerization during heating. This effect results in less char residue, despite the improved morphological stability and swelling observed for Al^3+^ beads.

The CMC_Al^3+^ reticulated beads also exhibited three degradation stages. The first *T*_max_ weight loss, occurring at around 60 °C, was attributed to the water outlet, while the others were attributed to CMC decomposition, as Al^3+^ ionic cross-linking of CMC and CMC partially substituted with Al^3+^ as counterions. The reported *T*_max_ values for CMC salts with aluminum as counterions are 197.53 °C, 272.19 °C, and 397.18 °C, respectively.^35^ On the other hand, the CMC_Ca^2+^ reticulated beads/conglomerates exhibited five degradation stages. This system’s complex degradation pattern is evident, where different CMC types coexist, including the original CMC salt, CMC partially substituted with Ca^2+^ as counterions, and diverse Ca^2+^-ionic cross-linked CMC. Liu et al. (2015)^33^ reported 305.67 °C as the *T*_max_ value for CMC salts with calcium as counterions.^4^

The DSC curves are displayed in Figure 4c. The DSC analysis provides complementary insight into the thermal events associated with ionic cross-linking in CMC-based networks. For the nonreticulated CMC (CMC_NR), the single exothermic peak at 290.91 °C is attributed to the primary decomposition of the CMC backbone through the auto-oxidation process, attributed to the presence of oxygen from the carboxyl group. The disappearance of the exothermic peak in the cross-linked samples suggests that interactions occurred between the carboxymethyl (−CH_2_COOH) groups of CMC and the Al^3+^ and Ca^2+^ ions, providing evidence for the occurrence of cross-linking. In contrast, CMC_Al^3+^ beads exhibited a distinct endothermic event at 171.84 °C, which is associated with the decomposition of hydroxyl groups or hydroxyl groups bonded to aluminum. At 386.71 °C, a peak is observed, which can be attributed to the disruption of ionic interactions between Al^3+^ and carboxylate groups, indicative of chain scission and polymer decomposition. Due to the trivalent nature and high charge density of Al^3+^, these ionic bridges act as multidentate coordination sites that require higher activation energy to be broken.

The CMC_Ca^2+^ system displayed a more complex DSC profile, with an initial endothermic peak at 75.98 °C associated with the release of bound and physically adsorbed water, followed by broader endothermic transitions in the 215–227 °C range. These events likely reflect the progressive disruption of weaker and more heterogeneous Ca^2+^-mediated ionic cross-links. The multiplicity of thermal events observed for CMC_Ca^2+^ suggests the coexistence of regions with different cross-linking densities, including weakly coordinated domains and non-cross-linked polymer segments. Furthermore, event at 309.29 °C is associated with degradation of the polymer. Overall, the DSC results are consistent with the morphological and thermogravimetric data, confirming that Al^3+^ promotes a more homogeneous and energetically stable ionic network in CMC beads, whereas Ca^2+^ leads to structurally heterogeneous systems with lower thermal coherence. This mechanistic interpretation reinforces the role of cation valence and charge density in governing the thermal response of ionotropically cross-linked polysaccharide hydrogels.

The swelling profiles are presented in Figure 5. The results obtained demonstrated that both CMC hydrogels exhibited the typical behavior of superabsorbent materials, with swelling values between 200% and 800%, attributed to the presence of hydrophilic functional groups (−COO^–^ and −OH) within the CMC structure. However, a superior performance was observed for the hydrogels cross-linked with Al^3+^ ions, which reached a water absorption value (*V*_ab_) of up to 780%. In contrast, those cross-linked with Ca^2+^ presented a significantly lower value, approximately 240%.

**Figure 5:**
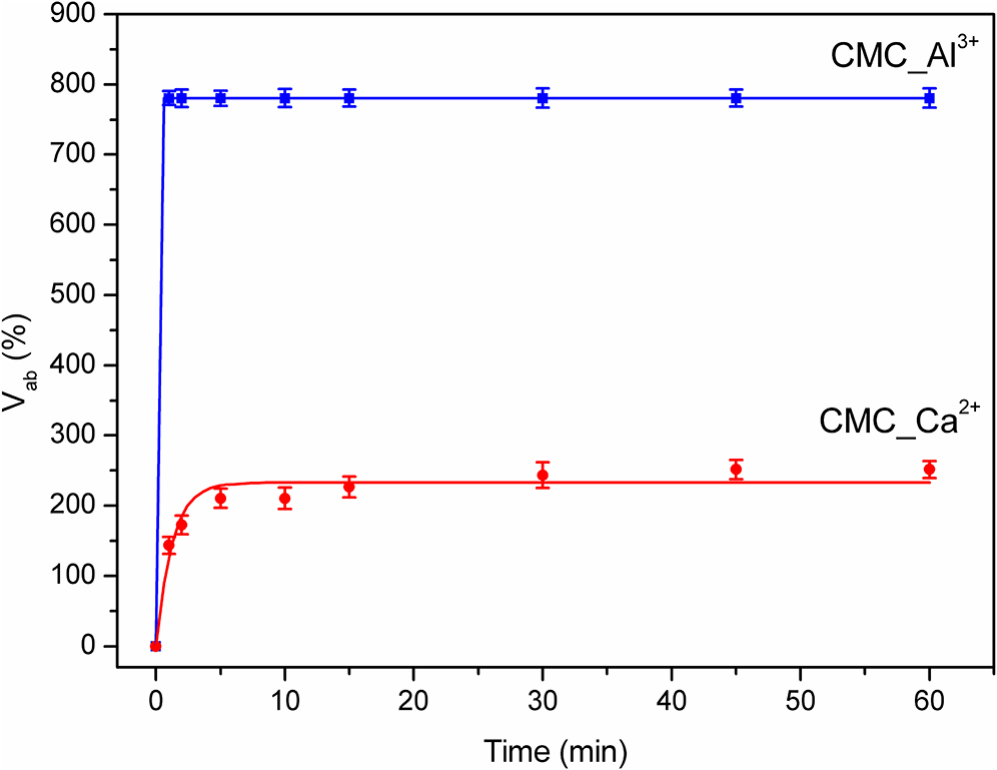
Swelling profiles of reticulated beads, represented by the absorbed water volume (*V*_ab_).

At first sight, this behavior appears to contradict the classical trend, in which higher cross-linking density typically leads to reduced swelling capacity. In our case, the trivalent Al^3+^ ion, due to its higher charge density and smaller ionic radius, not only forms stronger ionic bridges with the carboxylate groups of CMC but also promotes a more homogeneous and stable three-dimensional network. This architecture prevents collapse during drying and maintains interconnected pores that facilitate water uptake. In contrast, the rapid diffusion of Ca^2+^ ions tends to generate surface gelation and irregular conglomerates, producing heterogeneous and less porous matrices that restrict water penetration. Moreover, the Al^3+^ network likely retains a fraction of −COO^–^ groups not directly engaged in cross-linking, which enhances osmotic forces and hydrogen bonding, thereby favoring water absorption. Similar findings have been reported in other CMC and polyanionic hydrogel systems, where Al^3+^ cross-linking produced compact yet highly absorbent networks due to the coexistence of strong ionic bridges and residual hydrophilic groups.^33,34^

Although the actual concentration of incorporated cross-linking cations was not directly quantified in this study, multiple indirect pieces of evidence support distinct degrees of ionic incorporation and coordination in the CMC networks. FTIR spectra revealed the emergence of new bands at 1486 cm^–1^ and 1458 cm^–1^ for CMC_Al^3+^ and CMC_Ca^2+^, respectively, attributed to carboxylate–metal coordination, along with shifts in the asymmetric and symmetric COO^–^ stretching modes. These spectral changes indicate effective replacement of Na^+^ counterions by multivalent cations and the formation of ionic cross-links. Moreover, thermogravimetric analysis further supports this interpretation. The reticulated systems exhibited distinct degradation profiles and residual-mass behavior compared to nonreticulated CMC, reflecting modifications in the polymer–ion coordination environment. In particular, CMC_Al^3+^ beads showed fewer and more defined degradation stages, whereas CMC_Ca^2+^ samples displayed a broader and more complex decomposition pattern, consistent with heterogeneous coordination and partial cross-linking. These differences are consistent with previous studies^33,35^ reporting higher binding affinity and coordination density for trivalent Al^3+^ ions than for divalent Ca^2+^ ions in carboxylate-containing polysaccharides. The DSC suggested the cross-linking process, as CMC_NR shows an exothermic peak, indicative of the presence of carboxyl groups, whereas CMC_Ca^2+^ and CMC_Al^3+^ do not. The absence of the peak in these samples is due to the binding of the cations. Taken together, FTIR, TG/DTG and DSC results, combined with the pronounced differences in morphology, swelling behavior, and thermal response, strongly indicate higher effective cross-linking efficiency in the Al^3+^-reticulated CMC beads, despite the absence of direct metal quantification. In this context, analyzing all results together, it is evident that Al^3+^ ions favored spherical bead formation with uniform morphology and superior swelling, while Ca^2+^ ions led to irregular conglomerates and lower water absorption. Therefore, beads with the desired characteristics for subsequent encapsulation of *B. bassiana* were obtained using Al^3+^ as the reticulating agent.

### 2.2 Morphological Characterization of the Bead Formulation Containing Fungus

*B. bassiana* blastospores liquid culture was obtained with 2 × 10^9^ blastospores mL^–1^. Then, the blastospores’ liquid culture was combined with a CMC solution using a simple, fast, and efficient method. The formulation with fungus exhibited a similar visual appearance to the CMC_Al^3+^ beads, and its morphology was investigated using scanning electron microscopy. The SEM images of these formulations (Figure 6) revealed the formation of spherical-like capsules with a core–shell structure, where the shell layer wraps the core.^36^

**Figure 6:**
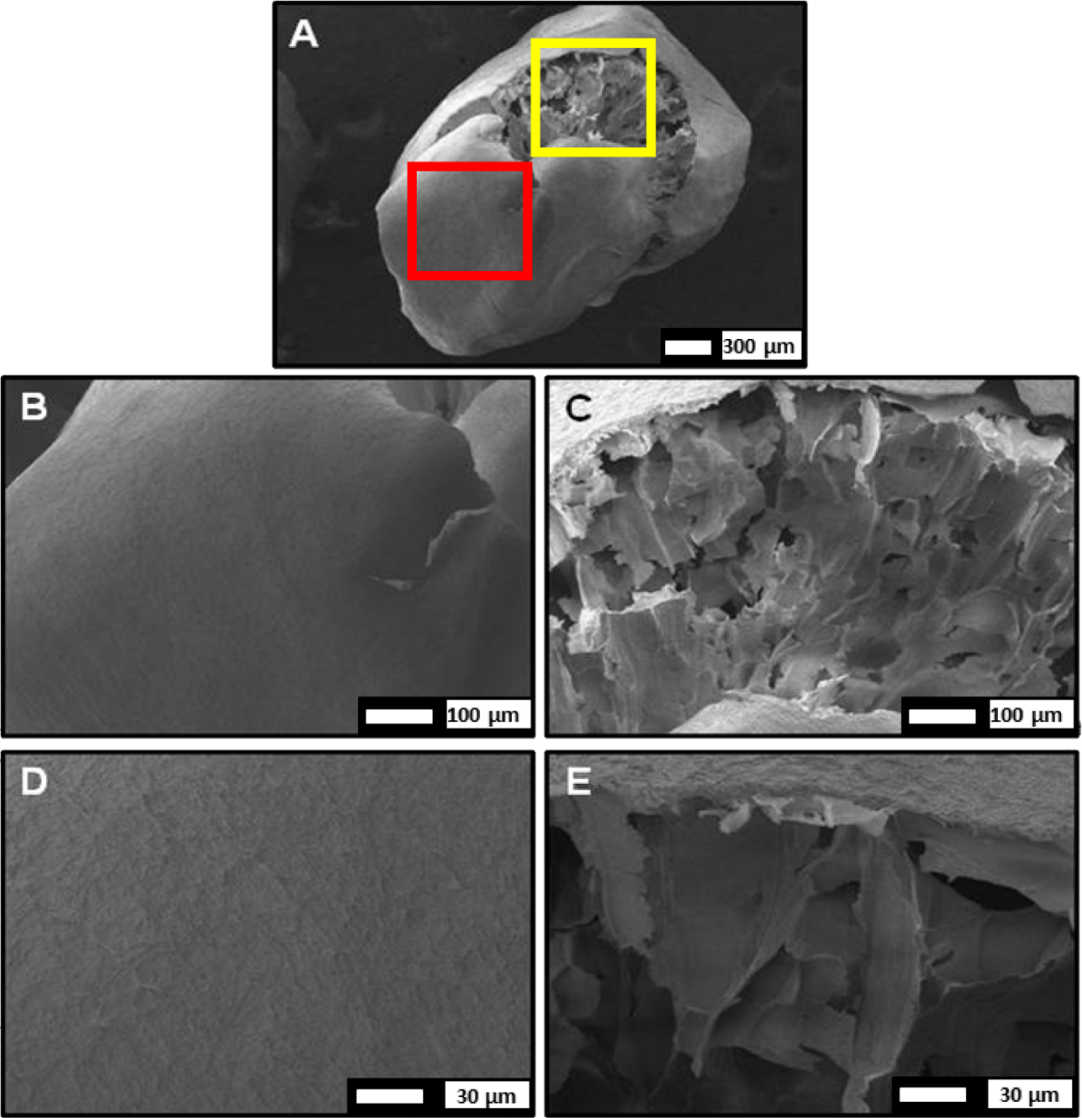
SEM images of a CMC granulate formulation reticulated with Al^3+^ and containing *B. bassiana*. (A) Capsule showing a core–shell structure; the red square marks the external shell region, and the yellow square marks the internal core. (B,D) Magnified views of the shell region, highlighting its thin, homogeneous, and slightly cracked morphology. (C,E) Magnified views of the core region, showing its irregular and porous structure.

The ionotropic reticulation of the CMC biopolymer forms the external surface of the capsule. The CMC shell layer is thin, homogeneous, and slightly rough with some cracks. The significant cracks permit the visualization of the inner part, the core. The core exhibits an irregular morphology with a sheet-like structure and disorganized, porous structures.^36^ It is formed by combining CMC, blastospore fungi solution, and Tween 80. The Tween 80 surfactants helped in the fungi-polymer dispersion.^24^

The morphology changes from beads to capsules due to the addition of fungi in the formulation. This fact can be explained by the heterogeneous dispersion of CMC in the hydrophobic fungal solution. Moreover, it was determined that the average size of CMC_Al^3+^ capsules containing *B. bassiana* was 2.42 ± 0.28 mm (mean ± SD, *n* = 50), indicating their increased size compared to the CMC_Al^3+^ beads (1.92 ± 0.12 mm, mean ± SD, *n* = 50). The *B. bassiana* blastospores were encapsulated in CMC beads to develop a dryable formulation with aerial conidia. Then, germination studies (Figure 7) were performed to evaluate the viability of aerial conidia on the bead surface after (A) two months and (B) five months of storage at −18 °C.

**Figure 7:**
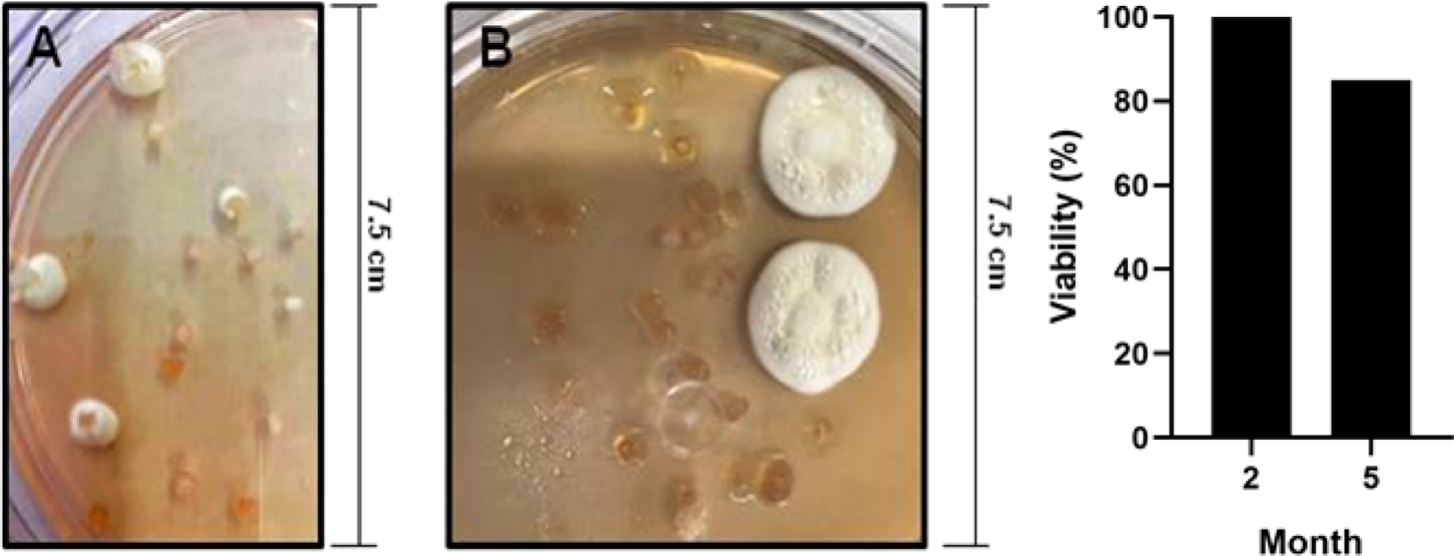
Digital images of fungus germination after two (A) and five (B) months of storage at −18 °C. Graphical of Viability of CMC_Al^3+^ beads after two and five months of storage at −18 °C.

During the study, the granules remained stable at −18 °C, maintaining their loose and very dry form after both two and five months of storage. Typical white aerial conidia of *B. bassiana* were observed on the granule surface after incubation, indicating successful reactivation of the encapsulated fungus. At two months, all granules germinated within 3–4 days, achieving 100% germination at a concentration of 1.87 × 10^6^ conidia per bead. After five months of storage, the granule formulation required 7 days to germinate. At this point, 85% germination was observed, with a concentration of 1.24 × 10^6^ conidia per bead. In contrast, nonencapsulated conidia stored under identical conditions exhibited only 69% viability after five months. The delay in germination observed after prolonged storage suggests a reversible metabolic dormancy rather than loss of fungal integrity, which is consistent with the protective role of the encapsulating matrix. The carboxymethylcellulose-based beads likely provide a buffered microenvironment that mitigates dehydration stress and limits oxidative damage during subzero storage, thereby preserving fungal viability.

Encapsulation of fungal propagules within biopolymers or inert matrices, such as diatomaceous earth, represents a viable strategy for the biological control of soil pests. The gradual release of conidia or mycelia into the environment enhances host infection efficiency and prolongs field activity.^37,38^ Moreover, it is well established that nonencapsulated propagules rapidly lose viability during storage, particularly under subzero or fluctuating temperature conditions. In contrast, encapsulation methods significantly extend shelf life by providing a protective microenvironment and maintaining a controlled moisture balance.^27^ In this study, encapsulation using carboxymethylcellulose increased fungal viability from 69% to 85% after five months of storage. Similar trends have been reported for *B. bassiana* encapsulated in alginate-based systems cross-linked with CaCl_2_, which showed improved resistance to temperature variations and ultraviolet radiation compared with nonformulated conidia.^39^ These results confirm that CMC-based encapsulation represents a promising formulation strategy for maintaining fungal viability during storage.

## 3 Conclusions

In this work, we prepared carboxymethylcellulose biopolymeric beads through ionotropic reticulation using Ca^2+^ and Al^3+^ ions. Then, for the first time, we demonstrated a feasible method for encapsulating *B. bassiana* blastospores in CMC_Al^3+^ beads. The CMC_Al^3+^ beads exhibited higher thermal stability, a uniform average size, and a greater swelling capacity than the CMC_Ca^2+^ beads. The encapsulation process was simple, fast, and efficient, resulting in aerial conidia on the bead surface (85% germination) after five months of storage at −18 °C. This result indicates that the encapsulation formulation strategy using the carboxymethylcellulose biopolymer has potential for biological control of soil pests.

## 4 Materials and Methods

### 4.1 Materials

Carboxymethylcellulose sodium salt (CMC, low viscosity, *M*_w_ ∼90,000) was supplied by Sigma-Aldrich. Calcium chloride (CaCl_2_) and aluminum chloride (AlCl_3_) were purchased by Êxodo Científica.

### 4.2 CMC Beads Obtention

Carboxymethylcellulose was dissolved in distilled water (2.0% weight/volume—w/v) under magnetic stirring at 60 °C for 2 h and cooled to 25 °C. Solutions of calcium and aluminum chloride (5% w/v) were prepared as reticulating agents. The CMC solution was injected into the reticulating agent using a syringe pump and stirred for 30 min. The obtained beads were washed with ultrapure water to remove unreacted AlCl_3_ or CaCl_2_ on the surface. The CMC beads were frozen and lyophilized at −50 °C using a LIOTOP lyophilizer. The samples obtained are identified as CMC_Al^3+^ and CMC_Ca^2+^, respectively.

### 4.3 Physical and Chemical Characterization

Fourier-transform infrared Spectroscopy (FTIR) was used to study the chemical changes of CMC gels by the reticulation process. The analyses were performed using a Cary 630 Agilent spectrometer in attenuated total reflectance (ATR) mode, with a frequency range of 4000–650 cm^–1^ and a resolution of 4 cm^–1^.

Thermogravimetric analysis (TGA) and differential scanning calorimetry (DSC) were performed in the range from 30 to 800 °C, at a heating rate of 10 °C min^–1^ and nitrogen with a flow rate of 100 mL min^–1^ (equipment SDT-Q600 TA Instruments).

The CMC beads were characterized by scanning electron microscopy (SEM) on a JSM-7500F SEM instrument (JEOL, Japan) to study their morphological characteristics. They were coated with carbon to render them electrically conductive and investigated at 2.00 kV to disclose their surface quality. The average diameter of beads was determined by optical microscopy (Zeiss, 400× magnification). Images of each formulation were analyzed with ImageJ software, and at least 50 individual particles were measured per sample. The mean values are reported along with standard deviations (mean ± SD).

The swelling studies were conducted using water absorption measurements on an Enslin device. The water uptake was determined by pouring approximately 0.05 g of reticulated CMC beads into the sintering filter and measuring the absorbed volume after predetermined times.^40^ The assays were carried out in quintuplicate, and the percentage of absorbed volume (*V*_ab_) was calculated according to eq 1, in which *V* and *w* correspond to the water volume at the time and the bead́s initial weight, respectively.

1 $$V_{ab}=\left(V_{water}/w_{initial}\right)\times 100$$

### 4.4 Culture, Growth Conditions, and Concentration Measurements of *B. bassiana* Blastospores

The *B. bassiana* blastospores were obtained by liquid culture at the Laboratory of Biological Control of the Biological Institute in Campinas, State of São Paulo, Brazil. The IBCB 66 strain was selected due to its pathogenicity against several pests with life stages in the soil, such as *Ceratitis capitata* (third instar, pupae, and adults),^22,41^ *Pratylenchus brachyurus* (eggs and second juvenile instar),^42^ *Spodoptera cosmioides* (third and fourth instar of caterpillar phase and pupae),^39^ *Chrysomya megacephala* (maggots in third instar),^43^ and *Atta sexdens rubropilosa* (workers and soldiers’ adults)^44^.

IBCB 66 strain is deposited in the Collection of Entomopathogenic Microorganisms “Oldemar Cardim Abreu”, held at this same laboratory, and stored in Eppendorf plastic tubes at −20 °C as pure conidia. The strain comes from the coffee berry borer, *Hypothenemus hampei* (Coleoptera: Scolytidae), collected in São José do Rio Pardo, São Paulo, Brazil.^24,45^

The fungal strain was inoculated for liquid culture work in Petri dishes containing potato dextrose agar (PDA) culture medium for growing. The Petri dishes were maintained in an incubator at 25 ± 1 °C, with a relative humidity (RH) of 70 ± 10%, and a 12 h photoperiod for 10 days. The produced conidia were removed from the PDA culture surface with a metal spatula, previously flambéed, to prepare the suspension in sterile 0.01% v/v Tween 80 (Sigma) aqueous solution. This conidia suspension contained 1 × 10^8^ conidia mL^–1^ and was used as an inoculum for the following liquid culture production.^45^

The liquid culture consisted of a combination of saccharose (40% C) and nitrogen in the form of yeast extract (23% N) per liter of medium, corresponding to a 10:1 carbon-to-nitrogen (C/N) ratio. It was poured into Erlenmeyer flasks containing 100 mL, sealed with a hydrophobic cotton cap covered by aluminum foil, and autoclaved at 1 atm for 20 min at 121 °C. Later, 1 mL of the produced conidia suspension was added to each Erlenmeyer and sealed inside the vertical laminar flow hood under sterile conditions. Then, the flasks were put to continuous agitation in a rotary shaker incubator (INNOVA 4000, New Brunswick Scientific, Edison, NJ, USA) at 40 rpm, 26 ± 1 °C, and in a 12 h photo period, in which they remained up to 8 days. Flasks were hand-shaken frequently to minimize mycelial growth on the flask wall.^45^

Samples were taken to measure the blastospore concentration in laminar flow at four, six, and 8 days postinoculation. One mL of these samples was added to 9 mL of sterile 0.01% volume/volume (v/v) Tween 80 aqueous solution as a wetting agent and mixed. The suspensions were quantified in a Neubauer chamber under an optical microscope (Zeiss) at 400× magnification. Two samples of 0.1 mL each were transferred to Petri dishes containing PDA culture medium to assess the quality of the fungus in terms of contamination. The plates were incubated at 25 ± 1 °C, 60 ± 10% relative humidity, and a 12 h photo phase for 7 days.^45^

### 4.5 Preparation of Fungal Encapsulation

The fungus encapsulation process was similar to the bead obtention, where the fungus was added to the cooled CMC dissolution, which had been previously autoclaved. Both solutions were stirred for 30 min before being dripped into the AlCl_3_ reticulating agent. 2.5 mL of blastospores liquid culture was used, and three drops of Tween 80 for each gram of CMC. All processes were achieved inside the vertical laminar flow, and all materials and reagents were first autoclaved for 30 min at 121 °C. After the freeze-drying process, the formulations were stored in a freezer (−18 °C).

### 4.6 Viability Test of the *B. bassiana*

Beads can function as microfermenters for encapsulated blastospores, facilitating the production of virulent aerial conidia.^46^ The viability of the fungus on the bead surface was assessed using 0.1 g of the formulation, which was weighed and placed on Petri dishes containing potato dextrose agar (PDA). Subsequently, 1 mL of distilled water was gently sprayed onto the beads to promote slow rehydration of the dried granules, minimizing osmotic stress and enabling the growth of the encapsulated *B. bassiana*. The plates were incubated at 25 ± 1 °C with a 12 h photoperiod for 18 h. After incubation, microscopic analysis was conducted to count the germinated and nongerminated structures in order to determine the percentage of viable conidia, as outlined in eq 2. Conidia were considered germinated when visible germ tube emergence was observed.

2 $$\%\;Viability=\left(N_{germinated}/N_{germinated}+N_{non-germinated}\right)\times 100$$

Viability assays were conducted after two and five months of storage at −18 °C. Nonencapsulated conidia maintained under identical storage conditions were used as control samples.

### 4.7 Concentration of the *B. bassiana*

The concentration of fungus on the bead surface was evaluated using the following methodology: (i) sprinkle 25 mg of the formulation on a PDA board and keep it at 27 °C for 4 days, (ii) wash the plate with 5 mL of sterile water, transfer to a tube, and add 5 mL of sterile 0.01% v/v Tween 20 aqueous solution. The number of existing conidia in the fungus suspension was counted with a Neubauer chamber, and the number of conidia produced per granule formulation was calculated.

## Supplementary Material



## Notes

Figure S1. Size distribution of CMC beads and capsules. Histogram showing the size distribution of both (a) Al^3+^and (b) Ca^2+^_crosslinked beads, as well as (c) the fungal capsules (PDF)
