# Supplementary material for: Sustainable Encapsulation of Biocontrol Agents: Cross-Linker Influence on Carboxymethylcellulose-Based Microbeads
Source: ACS Omega. 2026 Mar 13;11(11):17162–71. doi: 10.1021/acsomega.5c06970 (PMC13019400; doi:10.1021/acsomega.5c06970)
Supplement: Supplementary file 1 [file ao5c06970_si_001.pdf]

Sustainable Encapsulation of Biocontrol Agents: Crosslinker Influence on Carboxymethylcellulose-Based Microbeads

Mayté P. Zaldivar[1], Jean Carlos F. Machado[1], Lívia C. Massimino[1], Marcel S. Marques[2], José Eduardo M. de Almeida[3], Ana Paula S. Bartels[3], Ricardo Bortoletto-Santos[2], Hernane da S. Barud[1*]

[1] University of Araraquara (UNIARA), Biopolymers and Biomaterials Laboratory (BioPolMat), 1217 Carlos Gomes Street, Downtown – Araraquara, São Paulo, 14801-340, Brazil.

[2] University of Ribeirão Preto (UNAERP), Postgraduate Program in Environmental Technology, Avenida Costábile Romano, 2201 – Nova Ribeirânia, Ribeirão Preto, São Paulo, 14096-900, Brazil.

[3] Biological Institute, APTA/SAA-SP, Alameda dos Vidoeiros, 1097 – Sítios de Recreio Gramado, Campinas, São Paulo, 13101-680, Brazil.

Supporting Information

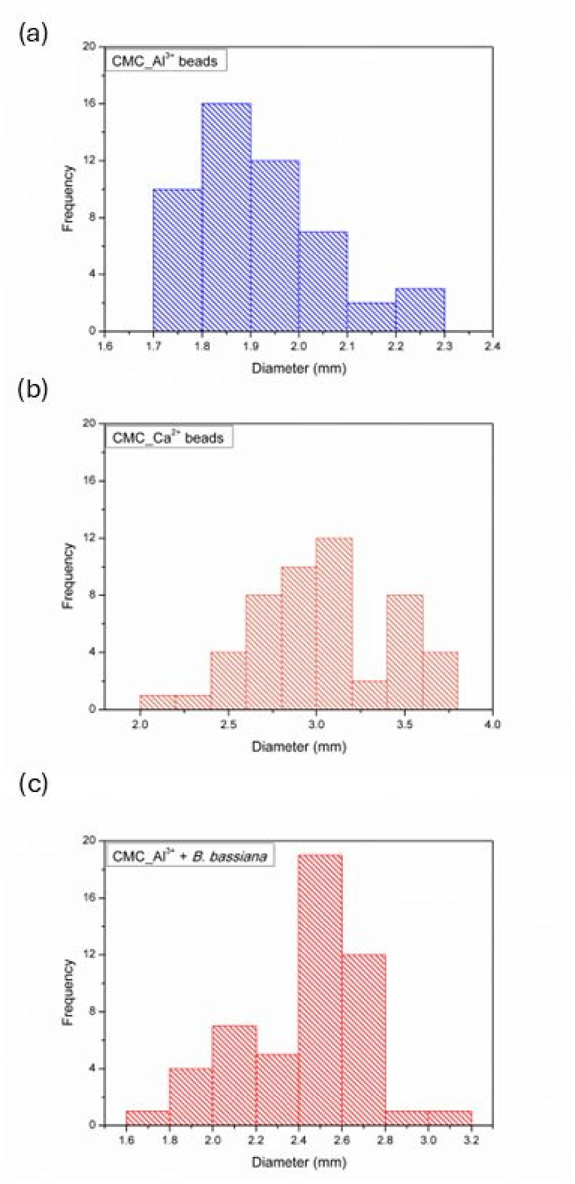

Figure S1. Size distribution of CMC beads and capsules. Histogram showing the size distribution of both (a) $Al^{3+}$and (b) Ca^{2+} _crosslinked beads, as well as (c) the fungal capsules.
